# A dataset of a proxy variable for the poultry trade flows in China

**DOI:** 10.1038/s41597-022-01793-6

**Published:** 2022-11-11

**Authors:** Lingcai Kong, Xuan Zhou, Mengwei Duan, Xinyi Yang, Caifeng Zhou, Yilan Liao

**Affiliations:** 1 North China Electric Power University, Department of Mathematics and Physics, Baoding, 071003 China; 2 Hebei Key Laboratory of Physics and Energy Technology, North China Electric Power University, Baoding, 071003 China; 3 Institute of Geographic Sciences and Natural Resources Research, Chinese Academy of Sciences, State Key Laboratory of Resources and Environmental Information System, Beijing, 100101 China

**Keywords:** Risk factors, Environmental sciences

## Abstract

Understanding the intercity poultry trading network is crucial for assessing the risk of avian influenza prevalence. Unfortunately, the poultry trading network in China has rarely been described. Here, with a modified radiation model, we obtain values for a proxy variable for poultry trade flows among 318 prefecture-level cities in China in 2015 utilizing the product capacity and demand quantity of poultry of the cities. The results are validated by comparing the proxy variable values with the trade volumes investigated in the literature, and it is found that the modified radiation model can accurately predict the main poultry trade flows among cities. This is the first dataset on China’s poultry trade pattern, and it can be used to analyze the production and consumption structure of poultry in prefecture-level cities within China. The dataset can be a tool for avian influenza epidemic risk assessment as well as a basis to develop prevention and control measures during an epidemic.

## Abstract (summary)

**Table:** 

Measurement(s)	poultry trade flows
Technology Type(s)	poultry food product
Factor Type(s)	poultry product • poultry consumption
Sample Characteristic - Organism	poultry
Sample Characteristic - Environment	poultry food product
Sample Characteristic - Location	China

## Background & Summary

The poultry trade plays a key role in the food supply, as poultry is one of the main sources of animal protein, including milk, meat and eggs. However, it is also believed to play an extremely important role in the spread of avian influenza, particularly over long distances and across different regions^1–3^. Therefore, understanding the trade patterns is of great help to develop avian influenza control strategies. Unfortunately, poultry trade flows in China have rarely been reported, except for a few local surveys^2,4,5^.

To estimate interregional trade flows, survey, non-survey, and hybrid methods have been widely used^6^. The survey-based approach can provide precise results but is not always available due to the large workload and high costs. Non-survey approaches, such as gravity models, entropy and information theory models, neural network models, and behavior-based models, provide alternative practical methods in the estimation of trade flows, despite some loss in accuracy^7^. Among the non-survey-based methods, the most commonly used is the gravity models, emerging from mainstream modeling frameworks in economics, for trade pattern analysis and empirical research in economic studies^8,9^. The gravity model belongs to the so-called spatial interaction models, which predict and explain the movement of population, traffic, commodities and information between origins and destinations in geographical space^10^. Despite its widespread use, to estimate bilateral trades with gravity model, previous trade flow data is necessary to fit parameters in the model formula^11^. The radiation model was one alternative model with no empirical data required. It was proposed to predict the population mobility and migration patterns, requiring only the population data in each location^11^. Due to its parameter-free nature, the radiation model can be applied in cases lacking previous flow data.

**Fig. 1:**
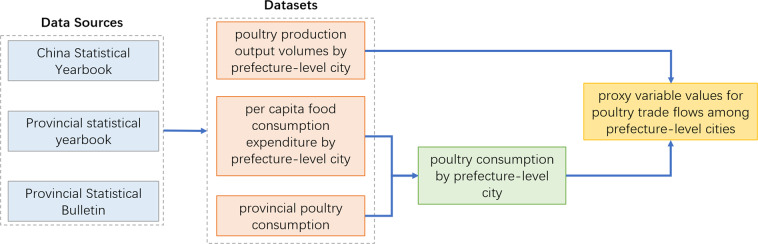
The whole data processing framework for poultry trade flow dataset production.

To fill the dataset gap in the field of poultry flows in mainland China, we adapted the radiation model, which was originally used to predict human mobility, and considered the production and consumption in each location the input to predict the interregional poultry trade flows. The whole data process framework is shown in Fig. 1. The results were validated with local survey data in the literature.

## Methods

### Data sources

To predict poultry trade flows, poultry production output volumes and consumption were needed. First, we used the slaughtered poultry volume for each prefecture-level city, collected from the statistical yearbook or bulletin (2016) of each province, autonomous region and municipality, as the proxy variable of the poultry production output volumes. Then, for poultry consumption, the per capita consumption of poultry by household at the province level was collected from the China Statistical Yearbook 2016^12^. To obtain the poultry consumption of each prefecture-level city, a downscaling method was applied, implemented by multiplying the provincial per capita poultry consumption of households by a weight, to reflect the differences between cities. The weight for city *i* was set as the ratio of the per capita food consumption expenditure of the city to the mean of the values in the province to which city *i* belongs. Finally, by multiplying the population size, the total poultry consumption of each city was obtained. The above indicators of cities were collected from the statistical yearbook or bulletin (2016) of each province.

### Original radiation model

The original radiation model was first presented by Simini *et al*. to predict population mobility and migration patterns^11^, which were assumed determined by a job selection process that consists two steps: (1) job seeking, assuming that the number of employment opportunities offered in each location is in proportion to the resident population there; (2) job selecting, individuals choose the closest job that offers benefits higher than the next-best offer available in the home location. Thus, distance has priority over benefits, as individuals are willing to accept more distant jobs only if there is a high enough benefit^13^.

Simini *et al*. also provided an equivalent description for the radiation model, using an analogy with the radiation emission and absorption processes in physical sciences^11^. The probability that *T*_*ij*_ particles emitted from location *i* (with population *m*_*i*_) are absorbed by another location j (with population *n*_*j*_) is a binomial distribution with average

1 $$\left\langle {T}_{ij}\right\rangle ={T}_{i}{p}_{ij}={T}_{i}\frac{{m}_{i}{n}_{j}}{({m}_{i}+{s}_{ij})({m}_{i}+{n}_{j}+{s}_{ij})}$$

and variance ${T}_{i}{p}_{ij}(1-{p}_{ij})$, where ${T}_{i}={\sum }_{j\ne i}{T}_{ij}$ is the total particles emitted by location *i*,

$${p}_{ij}=\frac{{m}_{i}{n}_{j}}{({m}_{i}+{s}_{ij})({m}_{i}+{n}_{j}+{s}_{ij})}$$

is the probability that a particle emitted from location *i* with population *m*_*i*_ is absorbed by another location j with population *n*_*j*_, given that *s*_*ij*_ is the total population in all locations (except *i* and *j*) within a circle of radius *r*_*ij*_ (the distance between *i* and *j*) centered at *i*.

### Modified radiation model

Here, as another analogy, we consider suppliers of poultry products in search of consumers as job-searchers, they choose closer consumers unless more distant consumers are willing to pay a sufficiently high price. In other words, we regard poultry been consumed as they obtained ‘work opportunities’. In this way, we can predict the poultry trade flows by modifying the original radiation model. In detail, we consider two locations *i* and *j* with production *m*_*i*_, *m*_*j*_ and consumption *n*_*i*_, *n*_*j*_, respectively, at distance *r*_*ij*_ from each other and denote with *s*_*ij*_ the total consumption in the circle of radius *r*_*ij*_ centered at *i* (excluding *i* and *j*). The average flow *T*_*ij*_ from *i* to *j* can be predicted by the radiation model Eq.(1) with ${T}_{i}={\sum }_{j\ne i}{T}_{ij}={k}_{i}{m}_{i}$, the total poultry production output to other regions from location *i*, where *k*_*i*_ is the proportion of exports to production in location *i*. In this study, we assume a constant *k* = 1 for all cities. In fact, any positive value for it provides proportional predictions of the proxy variable for poultry trade flows.

## Data Records

We produced two data products: the poultry production output and consumption data for each prefecture-level city in 2015 and the values for the proxy variable estimated for poultry trade flows among them. The dataset can be found in figshare^14^.

The data are stored in comma-separated values (CSV) format. The dataset on production and consumption contains the following fields:

**city –** The city included in the calculation.

**city_code –** The administrative area codes of the city.

**province –** The province to which the city belongs to.

**lon –** The longitude of the city.

**lat –** The latitude of the city.

**pop –** The population in the city in 2015.

**consumption –** The estimated total poultry consumption (value) in the city in 2015.

**product –** The estimated total poultry production (value) in the city in 2015.

The modified radiation model predicts the values for the proxy variable for poultry trade flows among each pair of cities. The dataset on these values contains the following fields:

**Origin –** The origin city of the flow.

**O.city_code –** The administrative area code of the origin city.

**O. Prov –** The province the origin city belongs to.

**Destination –** The destination city of the flow.

**D.city_code –** The administrative area code of the destination city.

**D. Prov –** The province the destination city belongs to.

**Flow –** The predicted values for the proxy variable for the poultry trade volume with the presented model from the origin city to the destination city.

**Prop –** The contribution rate of the proxy variable for poultry trade volume from the origin city to the total imports for the destination city.

**CumlProp –** The cumulative contribution rate of the proxy variable for poultry trade volume from the origin city for the destination city, calculated from largest to smallest for each origin city.

## Technical Validation

Few investigations of the poultry trade have been publicly released. Even so, we validated the estimation using investigated flows or statistical results published in available studies.

We first compare the origin cities exporting chicken to Guangzhou City with the investigation undertaken by Lin Hong *et al*.^4^. According to the investigation, chickens were the dominant avian species of live bird trade with a wide source, including 122 prefecture-level cities of 16 provinces of China. In addition to Guangzhou city itself, Yunfu, Foshan, Jiangmen, Zhaoqing, Qingyuan, Maoming and Shaoguan from Guangdong Province; Yulin from Guangxi Zhuang Autonomous Region; Nantong from Jiangsu Province; and Yuncheng and Jincheng from Shanxi Province were found to be the main sources exporting chickens to Guangzhou^4^. Obviously, most of them are located near Guangzhou, except Nantong, Yuncheng and Jincheng. We did regression analyses of the proxy variable for poultry trade flows with publicly available data on number and batches of chicken exporting to Guangzhou. Considering the different scales, we first normalized them to the range [0,1] with the formula

$${X}_{normalized}=\frac{X-{X}_{min}}{{X}_{max}-{X}_{min}}.$$

**Fig. 2:**
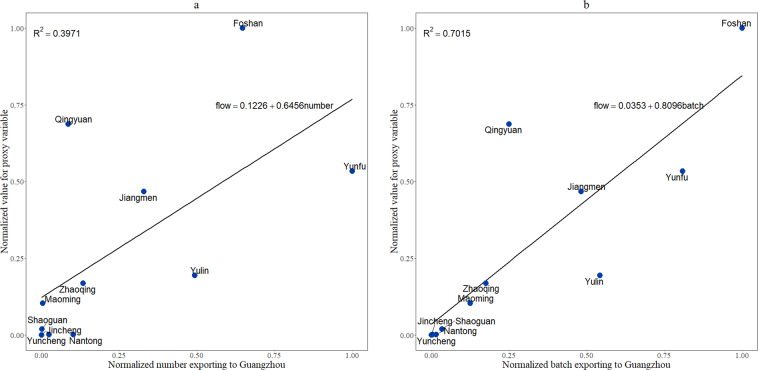
The regression results of the proxy variable for poultry trade flows with investigated number and batches from main sources exporting to Guangzhou.

The regression coefficients for the predicted proxy variable to the number and batches were 0.6456 and 0.8096, and with p-values of 0.0377 and 0.0013, respectively (Fig. 2). These results show that the actual flow data were positively associated with the proxy variable.

**Fig. 3:**
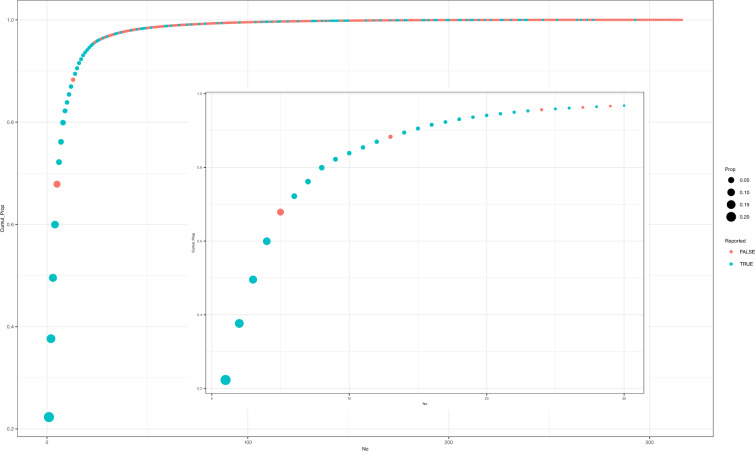
The cumulative rate of exportation to Guangzhou ordered by the volume predicted by the radiation model. The blue points represent cities that have been reported as exporting chicken sources to Guangzhou, while the opposite is true for the red points. The size of the points was determined by the predicted volume.

Now we compare the predicted sources of chicken exporting to Guangzhou with that had been reported^4^. First, we sorted the origin cities in descending order by the values for the proxy variable for poultry trade flows predicted with the presented radiation model. Then, the contribution rates and cumulative contribution rates for the total proxy variable value exported to Guangzhou were calculated. Table 1 shows the top 30 sources exporting poultry to Guangzhou according to our prediction. All the main chicken sources near Guangzhou were accurately predicted as the main sources exporting poultry to Guangzhou, except for Shaoguan, which was not included in our estimation because of missing data. Figure 3 shows the cumulative proportion of the values for the proxy variable for poultry trade flows exported to Guangzhou. Among the top 30 sources, 25 (83.3%) have been reported as chicken sources^4^. Therefore, the presented modified radiation model predicted the main sources exporting poultry to Guangzhou.

**Table 1:** The top 30 sources exporting poultry to Guangzhou predicted by the presented modified radiation model.

Origin	Values for proxy variable for trade flows predicted	Proportion to total columns to Guangzhou	Cumulative proportion to total columns to Guangzhou	Reported as sources by Lin *et al*.
Foshan	114268.7	0.223071	0.223071	TRUE
Qingyuan	78539.65	0.153322	0.376393	TRUE
Yunfu	60975.59	0.119034	0.495428	TRUE
Jiangmen	53338.82	0.104126	0.599554	TRUE
Nanping	40481.61	0.079027	0.678581	FALSE
Yulin-Jx	22127.38	0.043196	0.721777	TRUE
Huizhou	20305.16	0.039639	0.761416	TRUE
Zhaoqing	19249.77	0.037579	0.798995	TRUE
Maoming	11754.45	0.022947	0.821941	TRUE
Guilin	8503.421	0.0166	0.838541	TRUE
Heyuan	7992.58	0.015603	0.854144	TRUE
Dongguan	7894.495	0.015411	0.869555	TRUE
Chongqing	6889.456	0.013449	0.883005	FALSE
Meizhou	5871.328	0.011462	0.894467	TRUE
Qinzhou	5600.758	0.010934	0.9054	TRUE
Hengyang	5173.651	0.0101	0.9155	TRUE
Ganzhou	3856.188	0.007528	0.923028	TRUE
Shanwei	3852.58	0.007521	0.930549	TRUE
Jieyang	2875.017	0.005613	0.936161	TRUE
Longyan	2462.79	0.004808	0.940969	TRUE
Zhanjiang	2311.45	0.004512	0.945481	TRUE
Shaoguan	2133.053	0.004164	0.949645	TRUE
Nanning	1839.922	0.003592	0.953237	TRUE
Shantou	1584.008	0.003092	0.95633	FALSE
Ji-an	1215.081	0.002372	0.958702	TRUE
Changde	1157.966	0.002261	0.960962	TRUE
Chenzhou	961.7035	0.001877	0.96284	FALSE
Guigang	867.1939	0.001693	0.964532	TRUE
Changchun	858.2947	0.001676	0.966208	FALSE
Zhongshan	702.2188	0.001371	0.967579	TRUE

Since very few investigations on the poultry trade flows in 2015 are available, we compare some statistical results in the years around 2015 with our estimation. First, we compare our estimation with the movements of live broilers out of and within Guangxi in 2017 analyzed by Tang *et al*.^5^. According to their analysis of the movements out of Guangxi, Guangdong Province received the most movements (77.5%), followed by Guizhou (9.9%) and Hunan (8.6%). In our estimation, the corresponding proportions are 68.58%, 1.31% and 13.15%, respectively. That is, two of the three main exporting provinces from Guangxi were successfully predicted.

Another study that investigated the live poultry trading network around Changsha city, Hunan Province, was also used for comparison with our estimation^2^. According to the study, of the live poultry exported to Changsha, approximately 64% were from cities in Hunan Province, and the remaining 36% were from Hubei, Henan, Guangxi, Jiangxi, and Guangdong Provinces^2^. For our estimation, approximately 71% were from Hunan, and the other main sources included Jiangxi, Fujian, Chongqing, Hubei, Guangxi and Guangdong Provinces. Except for Fujian and Chongqing, the other provinces were reported to be the main source provinces. For the provinces importing live poultry from Changsha, Hunan Province itself accounted for approximately 79%, while the remaining were exported to Hubei, Guangdong, Guangxi, Henan, Yunnan, Jiangxi, Zhejiang and Shanghai, although the last three provinces (city) accounted for only a small percentage^2^. In our estimation, in addition to Hunan Province, where Changsha is located, the aforementioned provinces were also the main destinations of poultry exported from Changsha, with ranks of 3, 4, 5, 8, 16, 2, 12 and 14, respectively, by the proxy variable values in descending order.

**Fig. 4:**
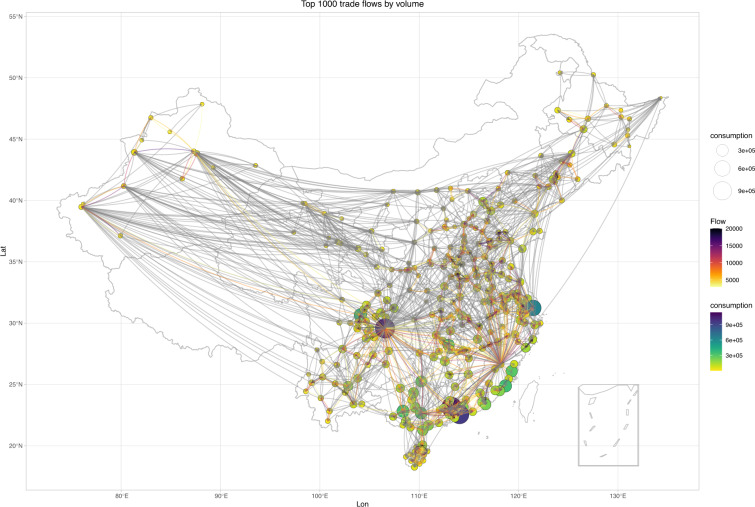
Poultry trade flows contributed 90% of the exports to each prefecture-level city predicted by the presented radiation model.

These comparisons show that the values calculated by the presented modified radiation model provide an accurate proxy for the main poultry flows among cities. What we need to do is to find a balance between keeping more flows and ensuring accuracy. Since a few sources contributed most of the poultry trade volumes^5^, we can use the contribution rates as threshold values to obtain the main sources for each city. Figure 4 shows the trade flows that cumulatively contributed to more than 90% of the total volumes for each city. These values can help analyze the production and consumption structure of poultry within China and can be used to assess avian influenza epidemic risk, which is the basis for developing prevention and control measures during an epidemic.

## Data Availability

The codes are fully operational under R 4.1.3. The package stplanr v0.4.0^15^ was referenced to implement the modified radiation; the visualization of the trade flows was implemented with the package sf v1.0-4^16^. The codes for the modified radiation model and the estimation are available on Github: https://github.com/MathGISer/Poultry-Trade-Network.
